# Local perceptions of mental health in Iran, Semnan Province

**DOI:** 10.1002/brb3.1971

**Published:** 2020-12-16

**Authors:** Monir Baradaran Eftekhari, Zohreh Mahmoodi, Masoumeh Dejman, Ameneh Setareh Forouzan, Katayoun Falahat, Mohsen Shati, Jafar Jandaghi, Jeremy Kane

**Affiliations:** ^1^ Deputy for Research and Technology Ministry of Health and Medical Education Tehran Iran; ^2^ Social Determinants of Health Research Center Alborz University of Medical Sciences Karaj Iran; ^3^ Department of Mental Health Johns Hopkins Bloomberg School of Public Health Baltimore MD USA; ^4^ Social Welfare Management Research Center University of Social Welfare and Rehabilitation Sciences Tehran Iran; ^5^ Mental Health Research Center School of Behavioral Sciences and Mental Health Iran University of Medical Sciences Tehran Iran; ^6^ Deputy of Health Semnan University of Medical Sciences Semnan Iran

**Keywords:** Iran, mental health, perception

## Abstract

**Introduction:**

Understanding local perceptions of mental health in different cultures and contexts is crucial for designing and implementing appropriate mental healthcare services.

**Methods:**

This qualitative study was conducted to investigate local perceptions of mental health in two highly populated provincial districts in Iran. Data were collected using the free list technique and interviews. A two‐phase training workshop was held with the research team at a local health center, followed by a pilot study with the participation of six subjects. All the interviews were audio‐recorded, transcribed, and then analyzed by the third and fourth authors in DEDOOSE.

**Results:**

A total of 30 individuals (20 in the free list and 10 as key informants in the interviews) took part in the study. Based on the study findings and the key informants' ideas, mental health problems were categorized into three categories of depression, anxiety, and obsessive–compulsive disorder (OCD).

**Conclusions:**

Mental health problems appear to be expressed in different ways and with different symptoms in different cultures, and there is a distinct need for examining mental disorders in each culture and nationality separately using culturally appropriate tools for disease screening.

## Graphical abstract

Given that the present study was qualitative, the results have a limited generalizability, and since the majority of studies on mental health have been quantitative, no further comparisons were possible. The present study was conducted in a defined population, which enables its continuation in the form of interventions and follow‐up of cases. Moreover, the study's use of the free list as a qualitative technique offers a model for similar future studies in this field.

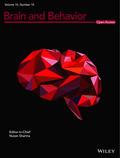

## 1 INTRODUCTION

Culture and context have profound effects on the perception of mental illness and paths that people follow in seeking healthcare help (Dejman, 2010; Dejman et al., 2010). Nonbiomedical beliefs about mental disorders and treatments are reported from all over the world (General & Services, 2001). It is widely believed that cultural groups differ on questions such as what is perceived to be a mental health problem (Fabrega Jr, 1991), and studies support the notion that culture and community play a central role in the emotional lives of people. Therefore, providing appropriate treatment and care in a community requires identification of the perceptions of mental disorder in that community and exploration and incorporation of culturally significant signs and symptoms of distress (Keyes & Goodman, 2006).

People's perceptions of illness influence their help‐seeking behavior or their failure thereof (Dow, 2011). Studies in different settings have revealed diverse beliefs on the subject, such as considering family conflicts as the cause of mental problems among Pacific Islanders (Douglas & Fujimoto, 1995) or seeing mental problems as an occasion for receiving a divine message in the Jewish population (Purnell, 2012). Similarly, in some cultures, mostly in South‐East Asia (Khan, Hassali, Tahir, & Khan, 2011; Mishra, Lucksted, Gioia, Barnet, & Baquet, 2009), as well as in some Western cultures (Pfeifer, 1994), people believe that supernatural forces are responsible for mental health problems.

There are cultural differences regarding the etiology of mental health problems and protective factors against them. For example, some Asian studies have shown that, in those societies, somatic and organic factors lead to emotional problems and medication is the treatment of choice (Naeem, Ayub, Kingdon, & Gobbi, 2012). In the Chinese culture, mental health problems and their etiology are explained as an imbalance of cosmic forces, and for treatment, it is preferred to use approaches that restore this balance through cognition (Zane, Takeuchi, & Young, 1994).

Using, without adaptation, standardized assessment tools developed in one culture may not accurately capture the psychological problems of another culture and can result in a distorted picture of the distress and the functioning of the patients. Similarly, imposing treatments developed in a different culture, with no consideration of local appropriateness, may be harmful and may increase distrust toward nontraditional practices (Okello & Musisi, 2006). Studies have shown that the perception of mental service providers as culturally insensitive plays a role in why people from non‐Western cultures are reluctant to refer to them for their and their family's mental health problems (Cauce et al., 2002).

Since the late 1980s, Iran has pursued the full integration of mental health care into the national primary care structure to establish a hierarchical referral system and provide better access to mental health services (Mohit, 2000). A study in 2006, after this integration, showed that the majority of mental health service users are treated in medical‐oriented outpatient clinics (948 per 100,000 of the population) and mental hospitals (130.4 patients per 100,000 of the population) (Organization, 2006). Despite these efforts, mental disorders have been among the most prevalent disabling disorders in Iran and, next to unintentional accidents, rank second in the list of the burden of diseases in the country. Recent studies have shown that increasingly more Iranians are suffering from mental disorders. According to a national survey in 2015, the prevalence of mental disorders was estimated to be around 23.44% (Noorbala et al., 2017).

In the last several years, an increasing amount of attention has been directed to the necessity of developing a culturally competent mental health delivery system in Iran (Forouzan et al., 2011). The present paper, as part of a large study, aims to describe the perceptions of mental illness among Iranians. We wanted to understand how Iranians perceive mental health problems and then use this information to explore whether Western‐described mental illnesses occur in this population and, if so, how they differ from their established descriptions. We plan to use this information on the local manifestations of mental illnesses to modify existing assessment instruments for local use. These adapted instruments would then be used in the future screening of mental illness and assessment of mental health intervention programs in this population.

## 2 METHOD

A rapid qualitative study using the free lists (FLs) and key informant (KI) interviews was carried out to explore how local people understand, describe, and prioritize mental health problems in their community (Ho, 2018). The qualitative data collection was the first phase in a large study and informed subsequent steps including the assessment of the highly ranked mental health problems, development of screening instruments, and planning of interventions. First, the free list technique was used to investigate the way local people briefly described the main mental health problems in their community. Then, interviews were conducted with key informants to understand how they conceptualized mental health problems in their community and to investigate in more detail the major mental or emotional problems described in the free lists and to explore whether symptoms “go together” and if there were any other significant symptoms that had not yet emerged.

Preliminary meetings were held with the vice‐chancellor of Semnan Health Center, director of Semnan Health Network, and director of Mental Health Department of the province to introduce the purpose of the study, give them a general description of the type of questions that would be asked and the kind of individuals/groups of people likely to be interviewed, and request their assistance in recruiting interviewers for the study.

Twelve psychologists working in urban health centers in Semnan and Garmsar districts were selected to interview participants using the free list technique. Before starting the interviews, a four‐day workshop was held in two phases in Garmsar Health Center by the research team for these psychologists. The major focus of the first phase of the training was on instilling an understanding of the principles and rationale of qualitative interviewing in the workshop participants, and the second phase was mainly concerned with teaching FL and KI interviewing techniques.

Following the workshop, a pilot study was conducted with six clients, three in Semnan and three in Grammar, to evaluate interviewers and assess the feasibility and applicability of the data collection techniques. A one‐day training event was held after the pilot study to review the positive and negative points of the pilot study and recruitment process and select interviewers for the main part of the study.

### 2.1 Setting

The study fields were Semnan and Garmsar, the two most highly populated districts in Semnan province. They also have the lowest proportion of immigrant residents in the province. Semnan province is one of 31 provinces of Iran and is located in northcentral Iran 216 km east of Tehran. The province covers an area of 96,816 square kilometers, and its population was 352,285 people and 36,298 families at the 2016 census. This province was selected because it provided suitable access to the study team and good cooperation from mental health staff and because of its low rate of immigration. In Semnan province, 24 urban health centers, 10 rural health centers, 14 urban health posts in the urban areas, and 72 health houses in rural areas provide primary health care to households. The prevalence of suspected cases of mental disorders in the province was 14.5% (15.8% among females and 13.1% among males) according to a national survey in 2017; about 20% of those studied experienced somatization (13.5% of males and 21.4% of females), 23.8% anxiety (17.7% of males and 2 6.8% of females), and 7.2% were suspected of depression (Noorbala, 2017).

Since 1986, the mental health program has been integrated into the primary healthcare system in Semnan province as part of The National Health Program, and this development has resulted in remarkable improvement in the provision of mental health services. The main components of the mental health program are advocacy, promotion, prevention, treatment, and rehabilitation. Since 2013, the integration of mental health has expanded by involving and hiring training psychologists in urban health centers.

### 2.2 Free listing

#### 2.2.1 Data collection

In the first stage of the study, the free list technique was used. Each interviewer had an Interview Guide Sheet to remind them of the interviewing steps and their order. Interviews had two sections. The first section included basic information about the participants such as age, educational level, marital status, and gender. In section two, respondents were asked “What are the main psychological or emotional problems that affect people in Semnan and could become targets of intervention and services?” They were prompted by the interviewer to list as many problems as possible and to give a brief description of each. The responses (i.e., problems) were recorded using the exact language of the interviewee (i.e., no summaries, paraphrasing, or translation) on a form comprising two columns. Responses to the primary question (i.e., the problems) were written in the left column. The interviewees were repeatedly probed for as many responses as possible until the interviewees indicated that they could think of no more problems. The interviewee was then asked for a brief description of each problem. This description was recorded in the right column of the form opposite the problem. The interviewer asked for one or two sentences only. If the interviewee responded with more sentences, the interviewer did not attempt to summarize the interviewee's comments but asked them to summarize their comments and recorded their summary.

#### 2.2.2 Study participants

Participants were selected from the general population with the assistance of women health volunteers (WHV) linked to Urban Health Centers and Health Posts. Invitation slips were distributed by WHVs in the population covered by health centers in Semnan and Garmsar districts. Households were asked whether they were interested in participating in the interviews for the study. The WHVs in the Urban Health centers contacted those who agreed to participate and scheduled a meeting where informed consent was obtained and free list interviews were conducted. Extreme case sampling was used to cover all target groups, including men and women above 18 years of age with diverse levels of education and marital status in different geographic areas. In total, 20 participants (11 men and 9 women) were included.

Community participants were also asked to identify individuals in their community who were commonly consulted by, or treated, people who were struggling with emotional or psychological problems. Their names and addresses in the cities were recorded for use in the next stage of the study. These individuals were approached later for the key informant interviews.

### 2.3 Key informant interviews

#### 2.3.1 Data collection

In‐depth interviews with key informants were used to understand the way informed individuals in the two districts conceptualized mental health problems in their community. The purpose of the KI interviews was to gather in‐depth information about the problems/symptoms selected. During the free listing, we found that the most commonly listed mental or emotional problems included many of the DSM‐IV diagnostic criteria for depression, anxiety, obsessive–compulsive disorder, and drug abuse. Therefore, during the KI interviews, the respondents were asked to sort the symptoms based on which symptoms “go together” and name each group. Then, they were asked whether they would add any other symptoms to the list. KI interviews started by describing each mental health problem/symptom that emerged from the free lists. They were then asked to name and describe all the problems that these symptoms might represent. Moreover, they were asked for any additional thoughts about the nature of each problem (e.g., what are its characteristics/symptoms or signs; how it is recognized).

#### 2.3.2 Study participants

Key Informants were selected either from among local individuals identified by free list interviewees or from a list, prepared by the director of the Mental Health Department, of the people who were potentially knowledgeable about mental problems. At the end of each interview, the interviewer asked the KI whether they knew of other individuals knowledgeable about the topic. The interviewer recorded the contact information of these people and they, too, were contacted and interviewed as KIs (snowball sampling). In total, ten people were interviewed.

### 2.4 Data analysis

After completing each FL and KI interview, data were immediately analyzed. All interviews were audio‐recorded and afterward transcribed and imported into Dedoose software and analyzed by the third and fourth authors. The results were provided as a summary sheet for each problem and the number of participants who had mentioned it or its equivalents. Then, the data were collapsed to build a composite list of problems ordered according to the frequency they were mentioned, thus providing an inventory of highly prioritized emotional and psychological problems.

A list of the free list symptoms that corresponded to the DSM‐V symptoms of depression, anxiety, and obsessive–compulsive disorder (OCD), along with symptoms that could not be categorized to any mental disorder, such as violence, anger, or addiction to the Internet, was constructed, representing highly prioritized mental problems, but given resource limitations and existing intervention programs for drug use disorder (DUD), we decided to concentrate only on depression, anxiety, and OCD.

This project has been approved by the Research Ethics Committee of the National Institute for Medical Research Development with ID: IR.NIMAD.REC.1395.047.

## 3 RESULTS

A total of 30 people (20 people in the free listing and 10 people in KI interviews) participated in the study (Table 1).

**TABLE 1:** Characteristic of participants in FL and KI interview in Semnan and Garmsar

Participants		Sex	Mean age	Education	Marital status
Free list	Garmsar	Men: 5 Women: 5	41.3	Under diploma: 4 Diploma: 7 Over diploma: 4	Married: 13 Widow: 2
	Semnan	Men: 6 Women: 4	39.3	Under diploma: Diploma: Over diploma:	Married: 10
Key informants		Men: 2 Women: 8	40.4	2 Psychiatrist 3 General physicians 3 Psychologists 1 High school Diploma	**–**

Of Garmsar and Semnan residents, 20 lay people were interviewed, 11 men and 9 women, in free list interviews. The most frequent mental health problems mentioned by study participants are included in a free list (Table 2).

**TABLE 2:** Results of the free lists mental health problems affecting residents in Semnan

Problem	Ranking	Number of respondents that include this problem
Anger/aggression	1	18
Loss of interest	2	16
Social withdrawal/Isolation	3	14
Scattered unexplained body aches	3	14
Impaired sleep	4	12
In constant worry	5	11
Irritability/Easily provoked	6	10
Feeling in blue	7	8
Lack of confidence and feeling badly about oneself	8	6
Too much thinking	8	6
Disturbing thoughts	8	6
Impaired/Low concentration	9	5
Low energy	9	5
Feeling restless/Restlessness	10	4
Thinking about suicide and self‐harm	10	4
Palpitation	10	4
Negativity/Pessimism	11	3
Feeling unsatisfied with almost everything	11	3
Repeated acts especially too much washing	11	3
Low religiousness	11	3
Lack of ability to deal with daily life problems	11	3
Feeling hopeless	12	2
Having a face with a sad expression	12	2
Excessive feelings of guilt	12	2
Crying too much	12	2
Eating too much	12	2
Inability to do daily tasks	12	2

In KI interviews, they were asked to sort the symptoms based on which symptoms “go together” and name each group (Table 3).

**TABLE 3:** Symptoms of mental health problems and related mental health disorders based on key informant's opinion

Symptoms of mental health problems	Name of mental health disorder
Loss of interest Social withdrawal/Isolation Impaired sleep Lack of confidence and feeling badly about oneself Low energy Thinking about suicide and self‐harm Negativity/Pessimism Feeling unsatisfied with almost everything Lack of ability to deal with daily life problems Feeling hopeless Excessive feelings of guilt Crying too much Inability to do daily tasks Eating too much	Depression
Scattered unexplained body aches Impaired sleep In constant worry Feeling in blue Impaired/Low concentration Feeling restless/Restlessness Palpitation Inability to do daily tasks	Anxiety
Scattered unexplained body aches Impaired sleep In constant worry Feeling in blue Impaired/Low concentration Feeling restless/Restlessness Palpitation Inability to do daily tasks	Obsessive–compulsive

The mental health problems were listed and prioritized into four problems. Based on the frequency and priority given to the problem/symptoms, depression, anxiety, OCD, and DUD were identified as common mental problems (Table 4). The research team could not categorize certain symptoms, such as violence, and addiction to the Internet, to any mental disorder to be explored in KI interviews. Given resource limitations and existing intervention programs for DUD, we later decided to concentrate only on depression, anxiety, and OCD. The findings regarding the definition and symptoms of each disease are presented later in the results.

**TABLE 4:** Common mental disorders and symptoms in Semnan

Common mental disorder	Symptoms' classification
Depression	Physical
	Cognitive emotional
	Social behavioral
Anxiety	Physical
	Behavioral
	Psychological
Obsessive–Compulsive disorders	Mental obsessions
	Behavioral compulsions

At the end of each interview, to confirm the descriptions of mental disorders that emerged from the free list and to obtain more detailed information about them, participants were asked directly to expand the definition of common mental disorders arisen from categorizing the free list content.

In this study, three main themes consisting of depression, anxiety, and OCD were extracted. There were two subthemes to each theme, including the definition of disease and disease symptoms, as follows:

### 3.1 A: Depression

#### 3.1.1 Definition of the disease

Most participants grouped mental problems under the heading of “depression” based on the symptoms and side effects of the disorder and, additionally, explained it through the symptoms that affect the function of sufferers.

#### 3.1.2 Disease symptoms

The depression symptoms mentioned by participants can be classified into the three groups of physical, cognitive–emotional, and social–behavioral symptoms.

Physical symptoms included low energy, lack of, or decrease in, sexual desire, and changes in appetite and sleeping habits.

Based on the participant's experiences, the most common physical symptoms were low energy and unexplained sporadic pain.

I had a headache, radiating to my shoulder. I feel tired. 40‐year‐old female, married, housewife, third‐grade literacy level.

Lack of, or decrease in, sexual desire was mentioned by one of the professionals interviewed in the study.

This symptom (decrease in sexual desire) is rarely addressed by patients due to the feeling of shame. People are ashamed to reveal something such as sexual problems related to depression.

Changes in facial expression, in the form of making a long face and looking gloomy and sad, were among the other symptoms mentioned by participants.

Changes in appetite and sleeping habits were considered as physical symptoms by participants.

His appetite is decreased; his sleeping habits are changing. He sleeps during the day, and, at night, he sleeps only after the dawn prayers. He is always saying that he is sleepy. I found out that he had a problem. 57‐year‐old female, married, high school diploma.

The most common symptoms described by the participants are classified as cognitive‐emotional. Among this group of symptoms, the most common complaint mentioned by both specialists and the general population was the loss of interest; most participants considered it a major symptom of depression.

I am not interested in doing anything; I feel down almost all days. I have no patience with my children. I am interested neither in food nor life nor my children. 40‐year‐old woman, married, housewife, third‐grade literacy level.

Other symptoms that were identified as depression symptoms by several of the participants were feeling displeased with oneself and others, particularly dissatisfaction with close relatives, worthlessness, and suicidal thoughts.

Some participants mentioned that religious beliefs play a protective role against suicide.

Thank god that suicidal thoughts were low among them. It is because of either their children or religious beliefs. 45‐year‐old female, married, physician.

Other symptoms mentioned included hopelessness and the feeling of guilt. Some participants from the general population considered concentration problems and inability to decide as symptoms related to hopelessness. Moreover, Participants mentioned feeling guilty about not performing a task they were supposed to do, even a long time ago.

Patients feeling guilty about divorcing from their sick spouse. 56‐year‐old female, housewife, B.A.

Symptoms of loss of confidence or low self‐esteem, feeling stressed, and feeling sad and unhappy (feeling the blues) were described by relatives of depressed patients.

In the social–behavioral symptoms group, four main subcategories were extracted, including lack of socialization, losing control of behavior, aggression and aggressive behavior, excessive crying and crying easily, and decline in function.

The most common symptom described by the participants was a lack of socialization. Most of the participants stated that this symptom occurred subsequent to the symptoms of impatience and irritability.

### 3.2 B: Anxiety

#### 3.2.1 Definition of the disease

Most participants grouped mental problems under the heading of “anxiety” based on the symptoms and side effects of the disorder.

#### 3.2.2 Disease symptoms

The anxiety symptoms described can be classified as physical, behavioral, and psychological symptoms.

Physical symptoms included palpitations, sweating, sleep disturbances, the feeling of choking in sleep, flushing, redness of the face, shivering, nausea, tingling sensations in the limbs, confusion, weakness, muscle stiffness, stuttering, headache, diabetes, hypertension, palms sweating, and sporadic pain.

Have noticeable stuttering, flushing, face redness, shaking in the hands and feet, and headache. 28‐year‐old male, lab technician.

Psychological Symptoms related to thought, such as irrelevant thoughts and thinking too much, along with the symptoms of the lack of concentration and difficulty in memorizing things, were grouped as anxiety symptoms.

Emotional symptoms included excessive worrying, irritability, aggression, feeling the blues, fear, constant anxiety, worrying over things such as unpleasant events, losing control of oneself, the fear that one might lose consciousness or memory, or faint.

Excessive thinking or nagging thought were common symptoms mentioned by most of the participants.

My patient thinks she may lose her child in a car accident or something unexpected like this. Then I feel palpitations and feel sick and restlessness. 40‐year‐old female, physician (GP).

Lack of concentration was considered as an intrinsic factor. “The patient has the capacity to perform a task but anxiety decreases his concentration and changes his mood.” 28‐year‐old male, laboratory technician.

Behavioral symptoms were grouped into three levels: individual, family, and social.

Making mistakes in daily activities, aimless struggling, restlessness, and aimless movements, a low threshold of tolerance for waiting, a tendency to rush things and repeat tasks, inability to perform daily tasks, and crying were examples of individual behavioral symptoms.

On the family behavior level, problems in interpersonal relationships, and aggression with children were stated by most of the participants.

Aggression, problems with work and social functions, struggling, impatience, getting easily upset if punctuality is not observed are examples of social‐behavioral symptoms.

### 3.3 C: Obsessive–compulsive disorder

#### 3.3.1 Definition of the disease

Most of the participants did not consider obsession as a disorder but described it as a personal trait.

Overall, two kinds of obsessions were mentioned, thought obsessions and practical obsessions.

In the urban area, thought obsessions were seen more often than practical obsessions and were very common, for instance, in the form of obsessive worrying about cheating or financial scams.

A good example of practical obsessions is the repeated checking of the gas valve.

#### 3.3.2 Disease symptoms

Symptoms that were addressed by participants in the study can be categorized into two major groups relating to practical and thought obsessions. The most common sign related to practical obsession, mentioned by both key informants and community members, was excessive and repeated washing.

People suffering from this symptom are always washing their hands or taking lengthy baths. Their hands are always wet, and they avoid touching things.

Some of the participants believed that excessive house‐cleaning is another symptom of these patients. They are constantly dusting and washing things, and the skin of their hands is damaged due to excessive dish‐washing.

Prolonged bathing takes from morning till afternoon. Married woman.

Excessive organizing and avoidance of being unorganized is another symptom. The sufferers over‐organize everything and loathe disorganization.

According to the participants, particularly the professionals, obsession limits the sufferers' social relationships because they are shunned by people on the account of their bothersome behavior, which is caused by obsession, or because the patients do not accept the others or trust them.

Participants stated that these patients do not venture outside their home and are exceedingly formal at home and that this problem can limit their socializing.

According to the experts and general population, repetitive behavior is the most common trait in obsessive individuals. They would not trust in the others' capabilities or relegate responsibilities but would try to handle everything themselves and, to feel comfortable, always need to repeat the tasks. After leaving their home, they are not sure whether they turned the gas off or locked the door. The symptom of repetitive behavior is reflected in their conversations as they constantly repeat the same story/talk and t also in their routines and activities, including religious functions.

Other signs mentioned include symmetry/mirroring obsessions, hoarding of useless things, and feeling guilty because of small mistakes.

Most professionals stated that obsessive individuals suffer from a lack of self‐esteem and think that they do not perform their tasks properly and, therefore, tend to re‐do them. Due to a lack of self‐confidence, they cannot make swift decisions and always doubt their decisions and try different patterns of decision making.

Based on the participants' opinions, signs of thought obsessions include troubling and recurring thoughts. The sufferers have worrying thoughts that torment them, for example, about food pollution.

He always thinks the food is polluted and can cause illness. 45‐year‐old male.

Bothering thoughts come to his mind which he does not like but cannot get rid of. 28‐year‐old male, laboratory technician.

## 4 DISCUSSION

According to the results, the symptoms of anger/aggression and loss of interest were considered as the first two priorities among the mental health problems by the people of the region. Based on the opinions of the important informed people, these symptoms were identified as part of the symptoms of depression and anxiety. Several studies have examined the relationship between anger and depression. A study by Luutonen (2007) revealed a direct connection between anger and depression, with anger or suppressed anger seen more in depressed patients. Furthermore, epidemiological studies indicate that depression is associated with a higher risk of violent behavior (Koh et al., 2002; Pan et al., 1994; Swanson et al., 1990).

Regarding the symptoms of depression, the results of the present study showed three general categories of physical, cognitive–emotional, and behavioral symptoms associated with this disease, with the most common symptoms related to the cognitive–emotional group, which includes the loss of interest. Other symptoms in this category include discontent with oneself and others, the feeling of worthlessness, and suicidal thoughts at the next level. Signs such as anger and aggression were also included in the group by the participants. The most common symptoms in the physical symptoms group were energy loss and sporadic physical pain, and in the behavioral–social group, social isolation, and aggressive behavior.

A longitudinal mediation analysis study, with data from the National Social Life, Health, and Aging Project (NSHAP), showed that individual isolation predicted higher depression and anxiety (Santini et al., 2020). Another study by Dilek et al. on depression symptoms was conducted using semistructured interviews. The results of the study indicated that signs of somatization, aggression, and violence were common symptoms of depression (Anuk & Bahadır, 2018).

Results of a systematic review of 138 studies in 170 populations of 76 different nationalities and cultures in 2017 indicated that overall, mood symptoms of depression/sadness, fatigue and lack of energy, and sleep disorder are three of the most important symptoms of depression reported not only in Western societies but also in other countries. However, some symptoms of depression, such as social isolation, excessive crying, anger and rage, general pain, and headache, which are not among the DSM‐V diagnostic criteria, are highly prevalent in depression in several countries (Haroz et al., 2017).

Based on the findings of the present study and according to the priorities set by the important informed people, mental health problems were categorized into three groups of depression, anxiety, and obsessive–compulsive disorder (OCD). Similarly, the qualitative study on the mental health problems of Somali Bantu and Bhutanese immigrants in Boston in 2014, indicated that conduct disorders, depression, and anxiety were the three most commonly reported mental problems in these two groups (Betancourt et al., 2015).

Most studies related to the mental problems around the world have suggested depression as the most important mental illness. For example, in a survey conducted by WHO in 2016 in 21 countries in 4 categories, 4 low/low‐middle income, 5 upper‐middle‐income, one low‐middle or upper‐middle at the times of two different surveys, and 11 high‐income countries, depression was the most common mental problem among the youth aged 18–22 years (Auerbach et al., 2016). However, a qualitative study done by Mohammadi et al. on the mental health problems among Iranian women showed that stress is causing persecution of Iranian women more than any other problem in the field of mental health (Mohammadi et al., 2014).

Anxiety and obsessive–compulsive disorder are the second and third priorities related to mental health problems in the current study. Regarding the prevalence of anxiety disorders, a study was conducted on 25,180 Iranian patients in 2005. The participants were selected using clustered random sampling, and the psychiatric disorders were determined based on criteria of DSM‐IV. The results of the study showed a prevalence of 8.35% for anxiety and 4.29% for mood disorders. Major depressive disorder and phobic disorder had the most prevalence among mood disorders and anxiety disorders, respectively (Mohammadi et al., 2005). The results of that study are not consistent with the finding of the present study; however, they indicate that anxiety and depression are two common psychological problems in Iran, both quantitatively and qualitatively. On the other hand, the results of the present study indicate that the third psychological priority is OCD. This finding differs from the results of Mohammadi's study, in which phobia was considered the most common anxiety disorder.

In 2020, a study was conducted on body dysmorphic disorder (BDD) and repetitive behavior. Based on DSM‐V, this disorder is a new category related to OCD. Both OCD and BDD cause marked distress and functional impairment in patients (Morgan, 2020).

It is noteworthy that, in the present study, levels of obsessions are the most important symptoms of the anxiety problem, and, at the same time, obsession itself is considered as one of the most important problems in the Iranian community. In most studies, obsessions have not been addressed as an independent disorder but as a sign of another disorder. In a study by Dawille et al. in 2016 on the susceptibility to OCD in Iranian, Lithuanian, and English adolescents, it was found that the Iranian subjects were more prone to OCD (Vore et al., 2016).

Mental health problems appear to be expressed differently and with different symptoms in different cultures, and there is a need for a deep examination of mental disorders in specific cultures and nationalities, as well as the development of tools appropriate to that culture allowing screening of these diseases properly. Any intervention to promote mental health primarily depends on the accurate and proper identification of mental health problems and then tailoring of the interventions to the culture of that community. To address this important issue, the present study tries to methodically probe common mental health problems in Semnan province, with the perspective of screening these problems in the community by constructing appropriate instruments, and ultimately, promoting mental health by designing interventions appropriate to the local culture. The results of the next phases of the study will be presented in later papers. Since this study was carried out qualitatively, it is not possible to generalize its results to other provinces of the country, which can be considered an important limitation of the study. However, the procedures used in this study can be used as a blueprint for promoting mental health by the health departments of medical universities throughout the country.

### 4.1 Limitations

Because of the qualitative nature of the study, its generalizability is limited and no further comparison was possible, as the majority of studies in the field of mental health are done quantitatively.

### 4.2 Strengths

Since this study was conducted in a defined population, it is possible to pursue it further by providing interventions and tracking cases. Furthermore, the use of free listing as a qualitative approach presented in this study could provide a model for other, similar studies on the subject.

## CONFLICT OF INTEREST

The authors declare that they have no competing interests.

## AUTHOR CONTRIBUTIONS

MBE has supervised the study, collected the data and statistical analysis, and wrote the manuscript. ZM contributed to the study design, collected the data, and conducted the analysis and wrote the manuscript. MD contributed to the study design, has supervised the study, analyzed the data, and drafted the manuscript. ASF contributed to the study design, has supervised the study, and drafted the manuscript. KF contributed to the study design, collected the data, and drafted the manuscript. MS performed the statistical analysis and drafted the manuscript. JJ and JK helped as Scientific Adviser. All author(s) read and approved the final manuscript.

### Peer Review

The peer review history for this article is available at https://publons.com/publon/10.1002/brb3.1971.

## Data Availability

The data that support the findings of this study are available from the corresponding author upon reasonable request.
